# Micro- and Nanoengineered Devices for Rapid Chemotaxonomic Profiling of Medicinal Plants

**DOI:** 10.3390/nano15120899

**Published:** 2025-06-10

**Authors:** Sajid Ali, Adnan Amin, Muhammad Saeed Akhtar, Wajid Zaman

**Affiliations:** 1 Department of Horticulture and Life Science, Yeungnam University, Gyeongsan 38541, Republic of Korea; drsajid@yu.ac.kr; 2 Department of Life Sciences, Yeungnam University, Gyeongsan 38541, Republic of Korea; adnan.amin@yu.ac.kr; 3 Department of Chemistry, Yeungnam University, Gyeongsan 38541, Republic of Korea

**Keywords:** chemotaxonomy, medicinal plants, microfluidics, nanoengineered sensors, lab-on-a-chip, surface-enhanced Raman spectroscopy, artificial intelligence, biodiversity conservation

## Abstract

Chemotaxonomic profiling based on secondary metabolites offers a reliable approach for identifying and authenticating medicinal plants, addressing limitations associated with traditional morphological and genetic methods. Recent advances in microfluidics and nanoengineered technologies—including lab-on-a-chip systems as well as nano-enabled optical and electrochemical sensors—enable the rapid, accurate, and portable detection of key metabolites, such as alkaloids, flavonoids, terpenoids, and phenolics. Integrating artificial intelligence and machine learning techniques further enhances the analytical capabilities of these technologies, enabling automated, precise plant identification in field-based applications. Therefore, this review aims to highlight the potential applications of micro- and nanoengineered devices in herbal medicine markets, medicinal plant authentication, and biodiversity conservation. We discuss strategies to address current challenges, such as biocompatibility and material toxicity, technical limitations in device miniaturization, and regulatory and standardization requirements. Furthermore, we outline future trends and innovations necessary to fully realize the transformative potential of these technologies in real-world chemotaxonomic applications.

## 1. Introduction

Chemotaxonomy—the classification of plants based on their chemical constituents—is an essential tool for accurately identifying medicinal plants [1]. This approach addresses key limitations associated with traditional morphological and genetic methods [2,3]. Traditional morphological classification is often challenged by phenotypic plasticity and environmental viability, leading to potential misidentification. Although genetic methods offer high precision, they are often expensive, time-consuming, and not well-suited for rapid, on-site analysis [4]. Consequently, research has increasingly focused on secondary metabolites—such as alkaloids, flavonoids, terpenoids, and phenolics—which serve as highly reliable chemical markers owing to their unique, species-specific, and relatively stable profiles [5,6]. These metabolites play essential roles in plant defense mechanisms and exhibit significant therapeutic properties, further highlighting their importance in identifying and authenticating medicinal plants [7,8].

Despite their analytical strengths, conventional methods for metabolite profiling—including high-performance liquid chromatography (HPLC), liquid chromatography–mass spectrometry (LC-MS), and nuclear magnetic resonance (NMR)—are limited by their lack of portability, speed, and user-friendliness in field-based contexts [9,10]. These methods typically require complex laboratory setups, expensive instrumentation, and extensive sample preparation, making them impractical for real-time, on-site applications, especially in resource-limited settings. Consequently, demand continues to rise for faster, more accurate, and portable analytical techniques capable of delivering rapid and reliable chemotaxonomic profiling directly in the field, where immediate results are critical for effective decision making in herbal medicine, biodiversity conservation, and detection of adulterated herbal products [11,12].

Recent advancements in microfluidics, lab-on-a-chip systems, and nanoengineered sensors have provided promising avenues for addressing these traditional limitations [13,14,15]. These miniaturized devices offer rapid, highly sensitive, and selective detection of secondary metabolites at reduced costs while requiring significantly reduced amounts of samples and reagents. Furthermore, integrating optical and electrochemical detection methods—enhanced by nanomaterials, such as gold nanoparticles, nanowires, and nanostructured surfaces—has significantly enhanced analytical sensitivity, facilitating trace-level detection of metabolites in complex plant extracts [16,17]. When coupled with artificial intelligence (AI) techniques—including machine learning and data fusion algorithms that integrate metabolomic, genomic, and environmental datasets—these devices enable sophisticated real-time analysis, automated classification, and enhanced accuracy in plant identification [18,19]. Despite their potential, the widespread adoption of these innovative technologies faces several challenges [20,21]. Key challenges—including concerns over the biocompatibility and potential toxicity of nanomaterials, technical limitations in maintaining high analytical throughput and sensitivity during miniaturization, as well as the absence of comprehensive regulatory guidelines and international standards—must be systematically addressed [22,23]. Overcoming these obstacles requires ongoing interdisciplinary collaboration among researchers, technologists, regulatory bodies, and industry stakeholders to ensure these innovative technologies achieve widespread adoption and effectiveness in practical applications.

This review aims to provide a comprehensive analysis of recent advances in micro- and nanoengineered devices for the chemotaxonomic profiling of medicinal plants. This review is distinct from earlier works by its focus on the integration of AI-based data analysis with micro- and nanoengineered devices, enhancing data processing capabilities for real-time, field-based plant identification. The review is organized into three main sections: (1) device types, (2) detection methods, and (3) applications. The device types section discusses the various nano-enabled devices used for plant metabolite analysis, such as microfluidic platforms, lab-on-a-chip systems, and nano-engineered sensors. The detection methods section focuses on the integration of optical and electrochemical sensors, enhanced by nanomaterials, to improve sensitivity and specificity in detecting secondary metabolites. The application of these technologies for field-based applications in herbal medicine, plant authentication, and biodiversity conservation is also emphasized, setting this review apart from those focused mainly on laboratory applications. Finally, this review addresses challenges related to portability, sensitivity, and cost-effectiveness and discusses strategies to overcome these barriers.

## 2. Target Metabolites and Analytical Needs

Analyzing plant metabolites is essential for classifying medicinal plants through chemotaxonomy. This approach helps identify and distinguish plant species based on their chemical composition [24]. Medicinal plants are rich in diverse secondary metabolites, each contributing to their biological functions and therapeutic potential. Compounds such as alkaloids, flavonoids, terpenoids, and phenolics are common markers used to classify plants into specific families or genera [25]. The chemical composition of these secondary metabolites is important not only for identifying the plant species but also for understanding their medicinal potential, as many of these compounds exhibit well-documented pharmacological activities [26]. As the need for fast and accurate plant identification grows, efficient profiling of metabolites has become increasingly important in areas such as pharmacognosy, biodiversity research, and herbal medicine development [26]. Traditional plant classification methods, which rely on morphological traits or genetic markers, have inherent limitations, especially when plants exhibit similar physical characteristics or require rapid identification [27]. Therefore, profiling these key metabolites using advanced analytical techniques has become an integral part of modern chemotaxonomy.

### 2.1. Overview of Major Phytochemicals in Medicinal Plants

Medicinal plants contain various secondary metabolites that serve as key indicators of their pharmacological potential and their relevance in chemotaxonomy. Among the most significant groups are alkaloids, flavonoids, terpenoids, and phenolics [28,29]. Alkaloids are nitrogen-containing compounds with strong pharmacological effects. For example, morphine from the opium poppy (*Papaver somniferum*) is used for pain relief, and quinine from *Cinchona* bark is effective against malaria [30,31,32,33]. These compounds frequently form the foundation of traditional medicines and modern pharmaceuticals, highlighting their importance in chemotaxonomic classification of plants. Flavonoids are common in many medicinal plants and are well-known for their antioxidant, anti-inflammatory, and anticancer properties. Flavonoids typically consist of two aromatic rings connected by a three-carbon chain, and they contribute to the coloration, flavor, and therapeutic properties of numerous plants [34,35]. Terpenoids represent the largest and most diverse group of plant metabolites. These compounds include essential oils, cannabinoids, and steroids, all of which exhibit therapeutic properties, such as antimicrobial, anti-inflammatory, and anticancer effects [36,37]. Furthermore, phenolic compounds, including tannins and lignans, are primarily responsible for the antioxidant and anti-inflammatory properties of many plants. They also play a key role in the defense mechanisms of the plant against environmental stressors [38,39].

**Figure 1:**
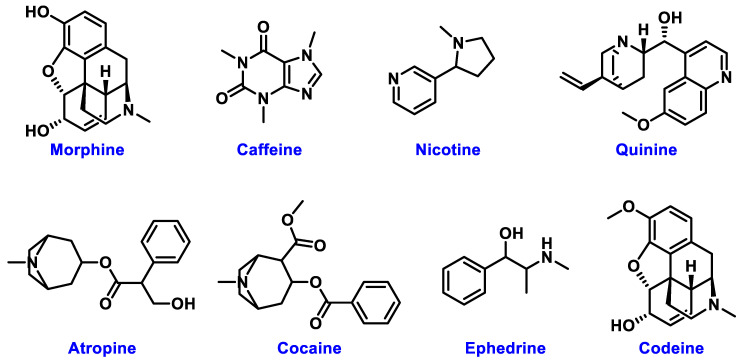
Structural Representation of Common Plant Alkaloids.

**Figure 2:**
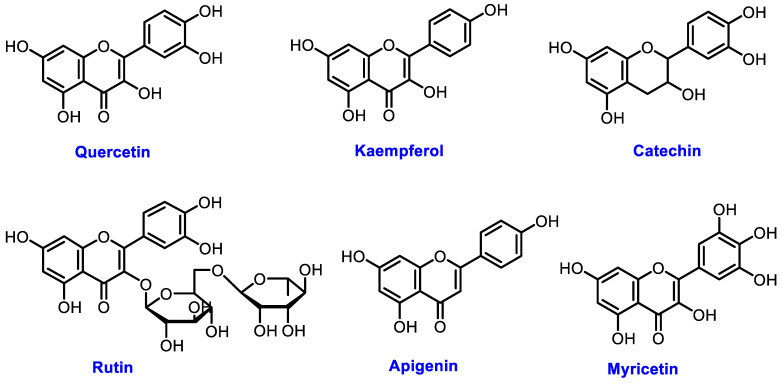
Structural representation of common plant flavonoids.

**Figure 3:**
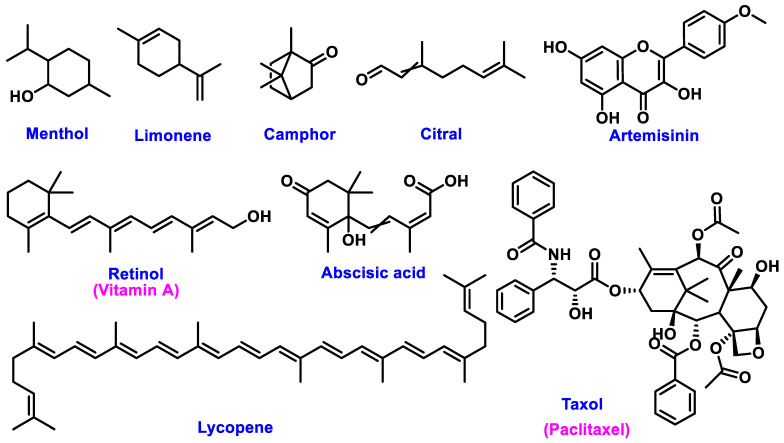
Structural Representation of Common Plant Terpenoids.

**Figure 4:**
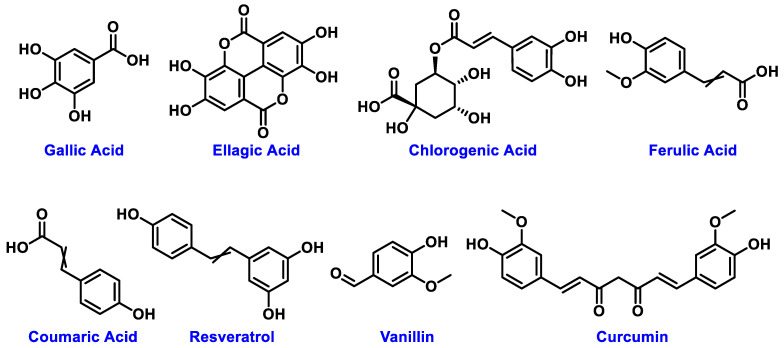
Structural representation of common plant phenolics.

**Figure 5:**
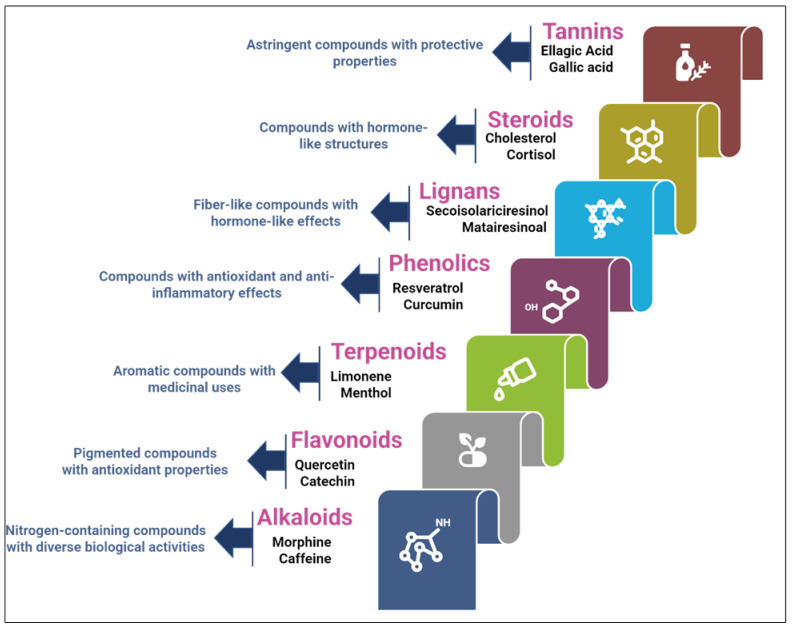
Representation of major phytochemical groups in medicinal plants.

The chemical structures of these metabolites are highly diverse, with each structure closely associated with its biological activity. Alkaloids contain nitrogen atoms within a heterocyclic ring and are often known for their potent effects on the human nervous system (Figure 1). Flavonoids have a basic 15-carbon skeleton and are further classified into various subgroups, such as flavones, flavonols, and isoflavones, based on their structural variations [40] (Figure 2). Their role in protecting plants from oxidative damage and their potential health benefits in humans have been extensively studied. Terpenoids are made up of isoprene units and show a wide range of structural complexity. They include simple monoterpenes like limonene and more complex compounds such as cannabinoids, which demonstrate various pharmacological effects [41,42] (Figure 3). Phenolic compounds consist of a hydroxyl group attached to an aromatic ring and include important subgroups like flavonoids, tannins, and lignans. These compounds contribute to several biological activities, including antimicrobial and anticancer effects [43,44] (Figure 4). Understanding the structural diversity of these compounds is essential for their application in chemotaxonomy, as they offer distinct chemical markers for differentiating plant species. Figure 5 illustrates a visual representation of the major groups of phytochemicals found in medicinal plants, showing types and examples from commonly used medicinal plants.

### 2.2. Challenges in Traditional Analytical Techniques

Despite the significance of secondary metabolites in plant classification and identification, traditional analytical methods, such as HPLC, LC-MS, and NMR, exhibit significant limitations, particularly in field-based or high-throughput applications [45,46,47]. HPLC remains a standard method for separating and identifying plant metabolites [48]. However, it is resource-intensive and requires advanced instruments, specialized reagents, and a stable power supply. These requirements pose challenges in remote areas where medicinal plants are often harvested [48,49]. LC-MS offers higher sensitivity and can analyze complex mixtures, but it also demands costly equipment and trained personnel, making it unsuitable for quick, on-site identification [49]. NMR, although highly effective for structural elucidation of metabolites, requires large sample quantities and expensive instrumentation. These limitations reduce its utility in field-based applications or real-time, high-throughput analyses [45,50].

Although traditional methods are highly effective in laboratory settings, they are not easily adapted for portable or on-site use. This creates significant challenges for applications in settings like herbal medicine markets or biodiversity conservation. As a result, there is an urgent need for compact and portable devices capable of performing rapid and accurate chemotaxonomic profiling without reliance on complex laboratory infrastructure. Emerging technologies such as lab-on-a-chip systems, microfluidics platforms, and portable spectroscopic tools offer promising solutions to address this challenge [51,52]. These miniaturized systems facilitate on-site analysis with precision comparable to those of traditional methods while offering advantages such as portability, low power consumption, and cost-effectiveness. By enabling chemical-profile-based identification, they can ensure accurate plant species identification even in remote locations [53]. Integrating AI and machine learning algorithms further enhances these systems by enabling the rapid and accurate processing of large, complex datasets. This advancement supports the development of real-time, high-throughput applications [54,55]. Consequently, developing portable and efficient analytical devices is essential to addressing the limitations of traditional techniques, enabling chemotaxonomic profiling to be conducted rapidly, efficiently, and accurately in field settings.

## 3. Microfluidics and Lab-on-a-Chip Platforms

Microfluidics and lab-on-a-chip platforms have transformed plant metabolite analysis by integrating sample preparation, separation, and detection into a single device. These miniaturized systems manipulate small fluid volumes through microchannels, offering significant advantages in speed, portability, and cost compared to those of traditional methods [13,52]. By consolidating multiple analytical steps on a single chip, microfluidics enables high-throughput, rapid, and on-site profiling of medicinal plants based on their chemical composition—which is crucial for chemotaxonomy [56]. The portability of these devices also makes them ideal for real-time, field-based plant identification and classification in remote areas.

### 3.1. Introduction to Microfluidic Systems

Microfluidic systems are designed to manipulate fluids at the microliter or nanoliter scale using microchannels that precisely control liquid movement. These systems rely on principles such as capillary action and pressure-driven flow to manage fluid dynamics within small-scale channels, often smaller than the width of a human hair [57,58]. A key advantage of microfluidics is its ability to integrate multiple laboratory functions—mixing, reaction, separation, and detection—into a single compact device. This integration enables high-throughput analysis with minimal sample and reagent consumption, making it highly efficient for plant metabolite analysis. The development of microfluidic systems has significantly affected fields such as clinical diagnostics, environmental monitoring, and chemotaxonomy, where rapid, cost-effective, and portable analytical techniques are essential.

**Figure 6:**
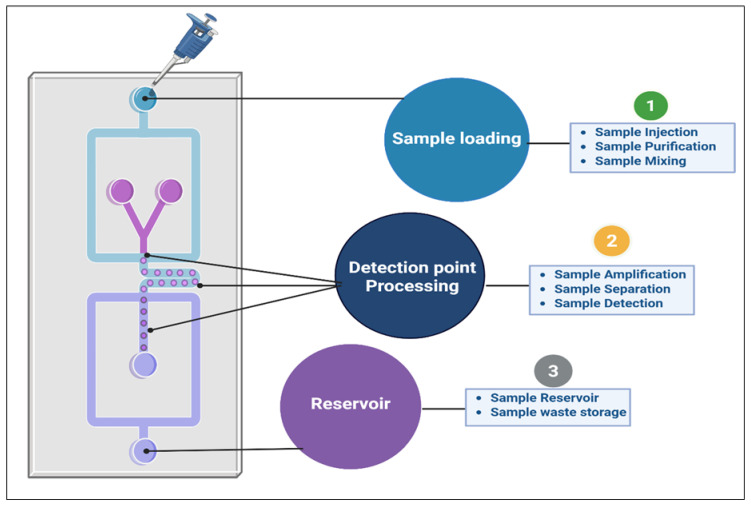
Schematic diagram of a lab-on-a-chip device for phytochemical profiling.

Miniaturization in microfluidics not only reduces analysis costs but also enables real-time monitoring and rapid processing of large numbers of plant samples. This is particularly valuable in chemotaxonomy, as it enables the rapid screening of multiple plant species based on their secondary metabolite profiles [14,59]. However, metabolite levels in plants can vary significantly throughout their life cycle due to growth stages, environmental conditions, and stress responses. This variability presents a challenge when using lab-on-a-chip systems, which are primarily designed for liquid samples. To overcome this, sample preparation methods such as liquid extraction, pre-concentration, and effective extraction techniques are necessary to ensure metabolites are present at appropriate concentrations for analysis. Furthermore, due to the structural similarities of metabolites such as alkaloids, flavonoids, and terpenoids, a single detection method may not be sufficient to discriminate and quantify them. To address this, multi-detection systems that combine fluorescence, electrochemical, and SERS techniques within a single platform are proposed. These combined methods can enhance sensitivity and specificity, improving the ability to distinguish between similar compounds. The integration of artificial intelligence (AI) for data fusion across these different detection techniques also improves the accuracy and efficiency of the plant metabolite profiling. These portable systems are especially well-suited for field applications, where traditional methods, such as HPLC or LC-MS, are impractical owing to their size, cost, and complexity (Figure 6).

### 3.2. Fabrication Techniques for Lab-on-a-Chip Devices

Lab-on-a-chip devices are commonly fabricated using materials such as polydimethylsiloxane (PDMS), which is preferred for its ease of use, optical transparency, and biocompatibility. PDMS-based devices are often produced using soft lithography, a rapid prototyping technique for microfluidic devices [60]. This process involves creating a mold using a photomask and then pouring PDMS over it to form microchannels, which can be used for separating and analyzing plant metabolites. While soft lithography is cost-effective and versatile, it has limitations, such as the potential absorption of certain small molecules and challenges in scaling up production [61,62]. An emerging fabrication method is three-dimensional printing, which enables the creation of custom-designed microfluidic devices by layering materials to build up the device structure of the device. This technique offers greater flexibility in design but may suffer from lower resolution than that of the traditional methods. Both fabrication techniques offer distinct advantages and limitations, depending on the specific requirements of chemotaxonomic profiling applications [63].

### 3.3. Integrating Detection Methods in Lab-on-a-Chip Systems

Lab-on-a-chip devices combine multiple detection methods for analyzing plant metabolites, making them versatile tools in chemotaxonomy (Table 1). Optical detection methods, such as fluorescence and absorbance, are widely used owing to their sensitivity and seamless integration with microfluidic systems. Fluorescence detection, for instance, is highly effective for identifying low-abundance metabolites by utilizing fluorescent tags or the natural fluorescence of the compounds [64]. Absorbance detection, typically used for compounds that absorb ultraviolet or visible light, provides reliable results for many plant metabolites, including phenolics and flavonoids. Electrochemical detection methods, in contrast, measure the changes in electrical properties caused by the oxidation or reduction of metabolites, offering high selectivity and sensitivity. These electrochemical sensors are particularly effective for detecting specific plant metabolites, such as alkaloids and terpenoids [65,66].

Real-world case studies reveal the successful integration of optical and electrochemical detection in lab-on-a-chip devices for metabolite analysis. For example, fluorescence-based lab-on-a-chip devices have been used by researchers to identify and quantify flavonoids in various plant species, while electrochemical sensors are employed to detect alkaloids in *Cinchona* bark [67]. These technologies offer rapid, low-cost, and portable solutions for field-based plant identification, which are crucial for herbal medicine markets, conservation efforts, and biodiversity monitoring.

**Table 1:** Comparison of lab-on-a-chip platforms for phytochemical analysis.

Detection Technique	Advantages	Limitations	Real-World Case Study	Examples of Plant Metabolites Detected
Fluorescence detection	High sensitivity for low-abundance metabolites. Non-invasive, rapid, and real-time detection. Easily integrated with microfluidic systems.	Requires fluorescent tagging or natural fluorescence. May not be applicable to all plant metabolites.	Fluorescence-based lab-on-a-chip devices are employed to detect and quantify flavonoids in plant species, such as Citrus and *Ginkgo biloba*, aiding in the identifying secondary metabolites in medicinal plants [68].	Flavonoids, phenolic acids, and anthocyanins
Absorbance detection	Simple, cost-effective, and widely used. Applicable to UV or visible light-absorbing compounds.	Lower sensitivity than that of fluorescence. May require extensive sample preparation for complex mixtures.	Commonly used to analyze phenolics and flavonoids, especially in agricultural and food safety applications. For example, used for polyphenol analysis in tea and grape samples [69].	Phenolic compounds, flavonoids, and tannins
Electrochemical detection	High selectivity and sensitivity for low concentrations. Suitable for real-time monitoring. Highly specific for certain compounds (such as alkaloids and terpenoids).	Requires specialized electrodes and systems. Limited to metabolites that can undergo redox reactions. Potential interference from other electroactive substances.	Electrochemical sensors integrated into lab-on-a-chip devices have been employed to detect alkaloids in Cinchona bark (for quinine) and terpenoids in aromatic plants such as lavender and peppermint. These sensors are particularly useful in herbal medicine research and conservation [70,71]	Alkaloids, terpenoids, cinchonine, and quinine
SPR	Provides real-time detection without labeling. Sensitive to changes in refractive index near the sensor surface. Non-destructive to samples.	Sensitive to surface conditions and requires highly specialized equipment.	Used in lab-on-a-chip devices to detect polyphenols and flavonoids by measuring refractive index changes at the sensor surface, often used for profiling complex plant mixtures [72].	Polyphenols, flavonoids, and antioxidants
CE	High resolution, fast, and effective for separating various metabolites. Can be combined with detection methods (UV, fluorescence, and electrochemical).	Requires more complex sample preparation and sophisticated equipment. May not be suitable for large-scale screening.	Used for separating and quantifying carotenoids and fatty acids in various plant extracts, especially in food quality control and metabolite profiling [73,74].	Carotenoids, fatty acids, and lipids

SPR, surface plasmon resonance; CE, capillary electrophoresis; UV, ultraviolet.

## 4. Nano-Enabled Optical and Electrochemical Sensors

Nano-enabled optical and electrochemical sensors significantly enhance plant metabolite analysis by utilizing the distinct properties of nanomaterials, such as nanoparticles, nanowires, and nanostructured surfaces [75,76]. These materials enhance sensitivity and selectivity, which are crucial for detecting low concentrations of metabolites, such as alkaloids, flavonoids, terpenoids, and phenolics. In optical methods such as surface-enhanced Raman spectroscopy (SERS), nanomaterials enhance performance by increasing surface area and enabling more efficient molecular interactions [77,78]. These nano-enabled sensors are typically portable, cost-effective, and well-suited for on-site applications. This makes them valuable tools in chemotaxonomy and medicinal plant identification, especially in field-based or high-throughput applications.

### 4.1. Introducing Nano-Enabled Sensors

Nano-enabled sensors function based on optical and electrochemical detection principles, where nanomaterials enhance the sensitivity and specificity of traditional methods (Table 2). In optical sensors, such as those based on fluorescence or absorbance, materials like gold nanoparticles, silver nanoparticles, and quantum dots amplify optical signals. This amplification makes it possible to detect plant metabolites at extremely low concentrations. For instance, gold nanoparticles are widely used in SERS. They significantly amplify Raman signals, enabling highly sensitive detection of trace compounds [79,80]. Electrochemical sensors, in contrast, measure current or voltage changes caused by redox reactions between metabolites and the sensor interface. Nanomaterials such as carbon nanotubes and gold nanowires are often employed to improve conductivity and signal transduction. These enhancements allow electrochemical sensors to detect specific plant metabolites with high precision. The progress in nanotechnology enables rapid, on-site analysis of plant metabolites, especially benefiting chemotaxonomy and identifying medicinal plants.

### 4.2. Surface-Enhanced Raman Spectroscopy for Plant Metabolites

**Figure 7:**
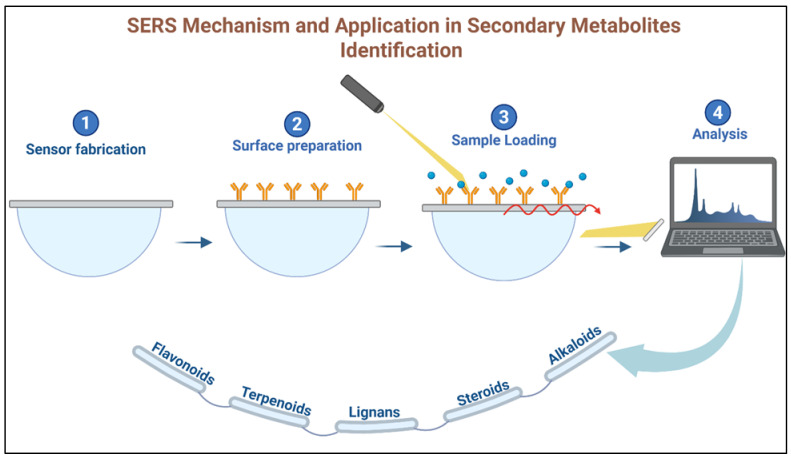
SERS mechanism and application in secondary metabolites identification.

SERS utilizes nanomaterials, typically metallic nanoparticles such as gold or silver, to enhance the Raman signal of plant metabolites. This enhancement arises from localized surface plasmon resonance, which occurs when metabolites interact with nanostructures, such as gold nanoparticle surfaces. The resulting signal amplification enables highly sensitive detection of low-abundance compounds within complex plant extracts [92]. SERS is particularly effective for identifying secondary metabolites, such as terpenoids, alkaloids, and phenolics, which are vital in plant chemotaxonomy. For example, SERS can be used to detect terpenoids in essential oils or alkaloids in plant extracts, providing valuable chemical markers for plant identification. Additionally, SERS requires minimal sample preparation, making it an efficient and non-destructive technique for real-time plant metabolite profiling [93,94]. Figure 7 illustrates the operating principle of a SERS-based nanosensor for plant metabolite detection, showing how the enhanced Raman signal enables compound-specific identification.

### 4.3. Field-Effect Transistor FET-Based Sensors for Phytochemical Detection

FET-based sensors are highly effective in detecting plant metabolites because they measure electrical changes that occur when metabolites interact with the sensor surface [95]. These sensors detect variations in current or voltage when specific molecules bind to the active surface of the sensor. Integrating nanomaterials, such as carbon nanotubes and gold nanowires, significantly enhances the sensitivity and selectivity of FETs for plant metabolites. Carbon nanotubes, for instance, offer excellent conductivity and a large surface area for metabolite adsorption, making FET-based sensors particularly efficient for detecting trace metabolites in plant extracts. FET-based sensors offer several advantages, including high sensitivity, miniaturization, and real-time operation, which are essential for on-site applications. They are particularly useful for detecting key metabolites, such as alkaloids and terpenoids, which are crucial for chemotaxonomy. Furthermore, their integration with microfluidic devices enables high-throughput analysis, making them valuable tools for large-scale plant metabolite profiling in the field [95].

## 5. Integration with Artificial Intelligence and Data Processing

Integrating AI and data processing with chemotaxonomy offers a transformative approach to plant identification [96]. Machine learning algorithms and data fusion techniques enable AI to analyze complex plant metabolic profiles alongside genomic and environmental data [97,98]. This integration enables rapid, accurate, and automated plant identification, enhancing the functionality of micro- and nanoengineered devices. AI-driven platforms can process vast amounts of data efficiently, offering high-throughput analysis vital for applications such as biodiversity conservation, herbal medicine, and counterfeit detection [98]. With ongoing advancements, AI systems are becoming increasingly capable of real-time, field-based plant identification, making the process more efficient and accessible than ever before.

### 5.1. Role of Machine Learning in Chemotaxonomy

Machine learning algorithms have become essential tools in chemotaxonomy, providing powerful methods to analyze and classify plants based on their metabolic profiles (Table 3). Algorithms such as neural networks, support vector machines (SVM), and decision trees are increasingly used to interpret the complex chemical data generated by advanced analytical techniques like HPLC, LC-MS, and SERS. These algorithms can be trained to recognize patterns in the chemical profiles of plant metabolites, allowing for accurate identification and classification of plant species [99]. Machine learning models are particularly effective at identifying non-linear relationships between metabolites, making them ideal for complex datasets with overlapping or intricate chemical signatures. Neural networks, in particular, are well-suited for handling large datasets and identifying non-linear relationships between metabolites, making them ideal for chemotaxonomy applications. Support vector machines, on the other hand, excel in classification tasks, especially when dealing with high-dimensional data such as plant metabolite profiles. By integrating these machine learning techniques, AI can significantly enhance the speed and accuracy of plant species identification, even with complex or overlapping chemical signatures [100,101].

### 5.2. Data Fusion Techniques: Integrating Metabolomics and Genomics

**Figure 8:**
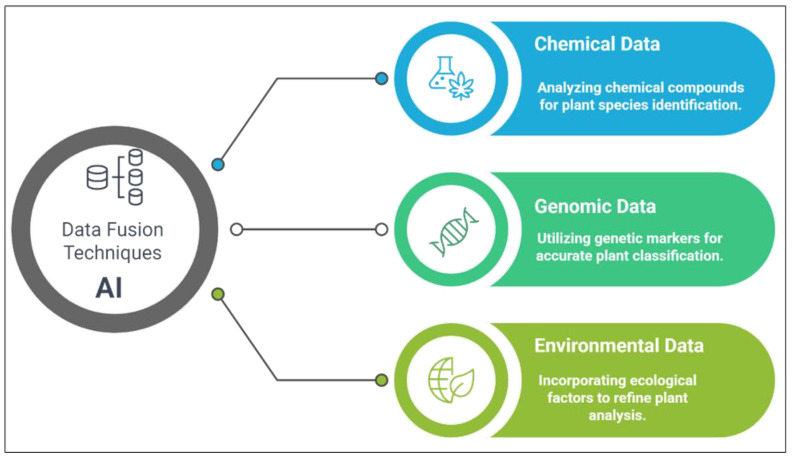
Data fusion techniques for plant identification.

Data fusion techniques integrate metabolomics with genomic and environmental data to enhance plant species identification (Figure 8). This integration enables more comprehensive insights into plant characteristics by leveraging their chemical and genetic profiles. For instance, combining metabolomic data with genomic markers enhances plant identification reliability by offering phenotypic and genotypic data points. This approach is particularly valuable when traditional morphological traits are insufficient to distinguish closely related species [114,115]. Incorporating environmental variables, such as soil conditions or climatic factors, allows AI systems to account for ecological variations that might influence metabolite profiles. These data fusion techniques enable the development of automated plant identification systems capable of processing and analyzing large datasets, thereby improving the efficiency and accessibility of plant classification.

### 5.3. Real-Time Data Processing for Field-Based Applications

Integrating AI with micro- and nanoengineered devices enables real-time, on-site data processing for field-based plant identification. These devices, often coupled with portable sensors and lab-on-a-chip platforms, can rapidly analyze plant samples based on their chemical profiles and deliver immediate identification results. Real-time data processing is particularly beneficial in remote areas with limited laboratory access, facilitating rapid, accurate in situ classification [116]. Additionally, developing portable, AI-driven plant identification platforms holds significant potential for enhancing the ability to monitor and conserve plant biodiversity, detect counterfeit medicinal products, and promote the sustainable application of plant resources. The potential for AI to process large datasets in real time and deliver immediate feedback is transforming the application of chemotaxonomy in research and field settings [117,118].

## 6. Applications and Case Studies

Nanoengineered devices have transformed plant metabolite profiling, with significant applications in herbal medicine, medicinal plant authentication, and biodiversity conservation. These technologies enable rapid, sensitive, and portable real-time plant identification, supporting the authenticity of herbal products and protecting endangered species [119]. Nano-enabled sensors, such as those based on SERS and FET technologies, have demonstrated high accuracy in plant species identification and adulteration detection, enhancing quality control and enabling efficient biodiversity monitoring [120].

### 6.1. Applications in Herbal Medicine

Nanoengineered devices are transforming herbal medicine by enabling precise detection of counterfeit herbal products and preventing species adulteration (Table 4). Their ability to detect subtle molecular differences is crucial for verifying the authenticity of herbal medicines [121,122]. By leveraging nanoscale technologies, these devices can distinguish between genuine herbal products from counterfeit or adulterated alternatives, ensuring the safety and efficacy of medicinal plant-based treatments. Given that counterfeit herbal products and species adulteration pose significant risks to public health, accurate detection methods are crucial. Nanoengineered devices enable molecular-level analysis of herbal products by detecting unique chemical signatures specific to each plant species. These devices, such as nanofluidic biosensors and molecular imaging tools, can detect trace levels of adulterants or fraudulent substitutions [123,124]. This rapid, accurate detection preserves the integrity of herbal medicine markets and safeguards consumer health [125]. For instance, Geetha et al. [126] demonstrate the application of nanoengineered devices to detect adulteration in ginseng products. Their study applied nanoparticle-based sensors to identify adulterants by comparing the chemical markers of authentic ginseng with those of counterfeit substitutes, providing a non-invasive, efficient method for quality control in the herbal industry.

### 6.2. Medicinal Plant Authentication

**Figure 9:**
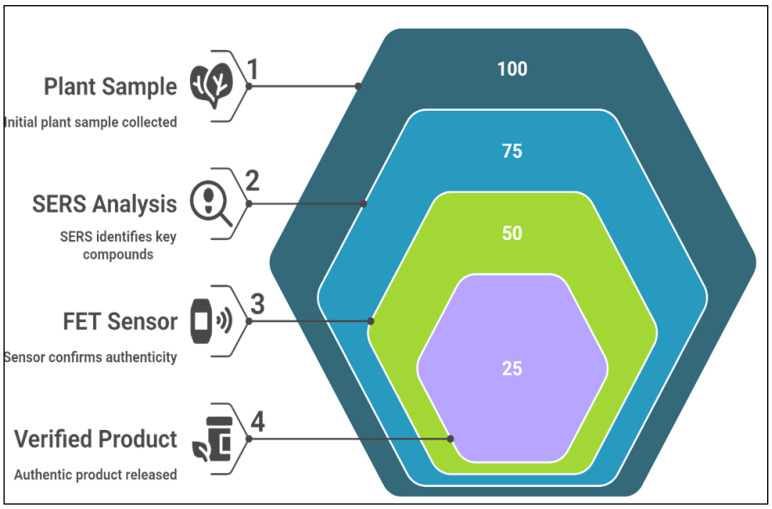
Process of medicinal plant authentication using nano-enabled devices.

Authenticating medicinal plants is crucial for consumer safety and species conservation (Figure 9). Nano-enabled devices offer efficient, cost-effective authentication methods. Benedetti et al. [134] demonstrated that SERS-based sensors could differentiate between *Ginseng* species, successfully distinguishing *Panax ginseng* from other species, such as *Panax quinquefolius*, based on their unique chemical profiles [135]. Similarly, Tavakoli et al. [136] employed FET-based sensors to authenticate *Withania somnifera* (ashwagandha), proving that nanosensors could accurately identify the plant, even within complex mixtures [137]. These studies highlight the crucial role of nano-enabled sensors in medicinal plant authentication and improving regulatory standards in the herbal medicine industry.

### 6.3. Biodiversity and Conservation Studies

Nanoengineered devices play a crucial role in biodiversity conservation by monitoring and protecting endangered plant species. Efficient, real-time plant species identification is crucial for assessing population health and directing conservation efforts on the right plants. Zhou et al. employed SERS-based sensors to monitor wild *Ginseng* populations, enabling non-invasive tracking of this highly valuable medicinal plant and aiding in its conservation [138]. This example highlights the crucial role of nano-enabled devices in real-time biodiversity monitoring and conservation of medicinal plants.

## 7. Challenges and Future Outlook

Despite significant advancements in micro- and nanoengineered devices for chemotaxonomic profiling, several challenges must be addressed before these technologies can be widely adopted in herbal medicine, plant authentication, and biodiversity conservation [122,139]. Current limitations include biocompatibility, material toxicity, technical reliability, and portability. Future research and development should focus on overcoming these barriers through innovative nanoengineering approaches, device miniaturization, and the integration of multi-sensor systems with mobile platforms. Additionally, addressing regulatory and standardization issues will ensure the broad acceptance and adoption of these technologies in chemotaxonomy and related fields [140].

### 7.1. Challenges in Current Micro- and Nanoengineered Devices

A key challenge in micro- and nanoengineered devices is ensuring biocompatibility and safety. Numerous nanomaterials, such as metallic nanoparticles or carbon-based nanostructures, may pose toxicity risks or interact adversely with biological systems, affecting human health and environmental safety. Concerns include nanoparticle accumulation, environmental contamination, and potential cytotoxicity, particularly in devices designed for repeated or direct plant contact, necessitating extensive safety testing and careful material selection [141,142].

Another major challenge is achieving consistently high throughput, sensitivity, and portability in nano-enabled analytical tools. Miniaturization often compromises sensitivity, limiting the performance of portable devices compared to those of laboratory-based instruments. Additionally, developing devices that can simultaneously analyze multiple metabolites within complex biological samples remains technically challenging [143]. To fully realize the benefits of nanoengineered chemotaxonomic profiling, future technologies must balance portability, sensitivity, robustness, and cost-effectiveness for effective field deployment.

### 7.2. Innovations in Nanoengineering for Field-Based Chemotaxonomy

Overcoming current limitations will require continued advancements in nanoengineering, particularly in device miniaturization, enhanced sensitivity, and multi-sensor integration. Emerging innovations include multifunctional “lab-on-a-chip” platforms capable of simultaneously detecting multiple metabolites or genetic markers, enhancing the accuracy and speed of plant identification [144]. Additionally, integrating these sensors with mobile devices such as smartphones or tablets further enables the development of portable, user-friendly, and cost-effective tools for field-based chemotaxonomic analysis [145,146].

Incorporating AI into nano-enabled systems also presents a significant opportunity for innovation. AI-based algorithms can rapidly process complex chemical data, enabling real-time, automated plant species identification even under challenging field conditions [76,147]. These innovations promise to deliver intelligent, miniaturized devices that can transform on-site plant authentication and biodiversity monitoring with broad applications in herbal medicine, agriculture, and conservation efforts.

### 7.3. Regulatory and Standardization Issues

With the growing application of micro- and nanoengineered devices in chemotaxonomy, addressing regulatory and standardization issues becomes crucial. Currently, regulatory frameworks for nano-based analytical devices are fragmented and often unclear, creating uncertainty for developers, manufacturers, and end-users. Regulatory bodies must establish well-defined guidelines for the safety, performance, and quality assurance of these devices to support commercialization and broad adoption [148,149]. In addition to regulatory issues, scalability remains a significant challenge. Transitioning from laboratory-based systems to large-scale, field-deployed devices presents obstacles related to cost, performance, and robustness. These devices must be miniaturized without compromising on sensitivity, which is essential for real-time, on-site plant identification in diverse environments. Furthermore, field validation is essential to ensure that nano-enabled devices perform reliably under variable environmental conditions such as temperature, humidity, and soil composition. Standardized testing protocols must be established to validate device performance across various environments and ensure consistency. Additionally, establishing globally recognized standards and validation protocols for plant identification using chemotaxonomic profiling is essential to ensure consistency, accuracy, and result comparability across devices and laboratories [150]. Standardized protocols will enhance the reliability of nanoengineered devices and support their acceptance by regulatory bodies, herbal medicine industries, and conservation organizations. This standardization is crucial for safeguarding consumers, advancing biodiversity conservation, and enabling the global adoption of effective nano-enabled plant authentication devices [151].

## 8. Conclusions

Micro- and nanoengineered devices are transforming chemotaxonomic profiling in medicinal plants by providing rapid and highly sensitive means of plant identification and authentication. Their potential to analyze complex plant metabolite profiles using innovative approaches—such as microfluidics, lab-on-a-chip systems, nano-enabled optical and electrochemical sensors, as well as integration with artificial intelligence—is advancing the development of more accurate, accessible, and efficient chemotaxonomic methodologies. These technologies facilitate real-time, portable, and high-throughput analyses, strengthening the reliability of medicinal plant identification in herbal medicine markets. They detect counterfeit and adulterated products and support biodiversity conservation by accurately identifying and monitoring endangered plant species. However, to fully harness the capabilities of these advanced technologies, several challenges must be systematically addressed. These include biocompatibility concerns, nanomaterial toxicity, and technical limitations in achieving optimal sensitivity, throughput, and balance between miniaturization and performance. Additionally, there is a pressing need for further integration of multi-sensor systems, AI-enabled mobile platforms, and data fusion techniques to improve real-time plant identification, particularly in field conditions. Clinical validation of these devices is also critical to ensure their accuracy and reliability under real-world, diverse environmental conditions. Innovations in nanoengineering, including multi-sensor system integration, enhanced miniaturization, and AI-enabled mobile platforms, present viable solutions. Continued innovations in these areas, along with standardized protocols for data interpretation, will be crucial for the effective deployment of these technologies in real-world applications. Equally crucial is the establishment of clear regulatory guidelines and globally standardized protocols for device validation and operation. Overcoming these barriers through coordinated research, industry collaboration, and regulatory oversight will facilitate the safe and effective adoption of these novel technologies across diverse real-world scenarios. Addressing the technical gaps in scalability, portability, and device integration will be essential to achieving widespread adoption. Continued advancements and interdisciplinary collaboration among scientists, engineers, regulatory agencies, and the herbal medicine industry will be crucial to advancing micro- and nanoengineered devices for accurate medicinal plant identification, public health protection, biodiversity conservation, and ecological sustainability. As these technologies mature and their adoption expands, they are poised to become indispensable tools, ushering in a new era of precision and sustainability in chemotaxonomic research and practice.

## Figures and Tables

**Table 2:** Types of nano-enabled sensors for medicinal plant profiling.

Sensor Type	Nanomaterials Used	Principle	Specific Applications	References
Optical sensors	Gold nanoparticles, silver nanoparticles, and quantum dots	They operate by amplifying light absorption or fluorescence with nanomaterials to enhance the signal.	Used in SERS to detect trace plant metabolites such as terpenoids and flavonoids.	[81,82]
Fluorescence sensors	Quantum dots, gold nanoparticles	Nanomaterials enhance fluorescence, enabling the detection of low metabolite concentrations.	Used to detect metabolites such as polyphenols and alkaloids in medicinal plants.	[83]
Electrochemical sensors	Carbon nanotubes and gold nanowires	These sensors detect electrical changes (current/voltage) caused by redox reactions when metabolites interact with the sensor surface.	Used for monitoring plant metabolites such as amino acids, vitamins, and neurotransmitters based on their electrochemical properties.	[84]
Electrochemical biosensors	Metallic nanoparticles and carbon nanomaterials	Integrate electrochemical sensors with biosensors to detect small biomolecules through surface interactions.	Applied to detect metabolites such as ATP, lactate, and glutamate, aiding in plant stress response and metabolite profiling.	[85]
Optical detection (SERS)	Gold nanoparticles and silver nanoparticles	SERS amplifies Raman scattering, facilitating the detection of plant metabolites at low concentrations.	Enhances the detection of secondary metabolites such as phenolics, terpenoids, and alkaloids, often used in chemotaxonomy for medicinal plant identification.	[86]
Nano-electrochemical sensors	Carbon nanotubes and silver nanoparticles	Detects changes in electrical properties induced by metabolite interactions with electrodes.	Provide rapid, on-site detection of plant metabolites with high sensitivity, such as polyphenols in medicinal plants.	[87]
Quantum dot sensors	Quantum dots and nanostructured carbon	Utilizes photoluminescence properties of quantum dots to detect specific plant metabolites.	Profiles secondary metabolites in plants, especially for identifying medicinal plant varieties.	[88]
Nanobiocompatible sensors	Chitosan nanoparticles and gold nanoparticles	Combines nanomaterials with biological molecules to improve selectivity and sensitivity.	Used for metabolite detection and profiling secondary metabolites in response to environmental stressors.	[89]
Biocomposite sensors	Silver nanoparticles and graphene oxide	Leverages the unique properties of carbon-based materials and silver nanoparticles to enhance metabolite detection.	Used to detect bioactive compounds, particularly for agricultural biotechnology and stress tolerance.	[90]
Multi-platform sensors	Graphene and silver nanoparticles	Combines various nanomaterials to create highly sensitive, multi-platform detection methods.	Applied in food safety for pathogen detection and in plant metabolite analysis.	[91]

SERS, surface-enhanced Raman spectroscopy; ATP, adenosine triphosphate.

**Table 3:** AI algorithms used for chemotaxonomic profiling.

Machine Learning Algorithm	Application in Chemotaxonomy	Strengths	Specific Use Cases	References
NN	Pattern recognition in large-scale plant metabolite datasets.	Capable of modeling complex datasets and recognizing nonlinear relationships.	Applied in plant classification based on their metabolic profiles, such as distinguishing species with overlapping chemical signatures.	[102]
SVM	Classification of plant species using biochemical data.	Well-suited for high-dimensional datasets and classification tasks.	Utilized in species classification, such as sweet oranges or Miscanthus, based on secondary metabolite profiles.	[103]
DT	Classifying plants based on metabolic markers.	Transparent and interpretable decision-making process.	Employed for identifying plant species, particularly effective for novel or rare species using chemical profile data.	[104]
RF	Improving classification accuracy in large and noisy datasets.	Ensemble approach that mitigates overfitting and improves model generalizability.	Classify and cluster plant species based on multifaceted metabolite data and environmental factors.	[105]
PCA	Dimensionality reduction for large chemotaxonomic datasets.	Simplifies complex data while preserving key variance.	Applied to simplify plant metabolite profile analysis and clustering of plant species.	[106]
KNN	Plant classification based on metabolite similarities.	Simple and effective for small to medium-sized datasets.	Applied in species classification by comparing chemical profiles and visual attributes.	[107]
LR	Binary classification for identifying specific plant traits.	Suitable for probability-based classification in binary scenarios.	Identifies specific plant diseases or traits based on metabolite data.	[108]
CNN	Image-based plant species and disease identification.	Well-suited for image recognition tasks and extracting spatial features from plant images.	Applied in real-time identification of plant disease and species from leaf images.	[109]
Capsule networks	Image-based classification, particularly for plant diseases.	Effectively captures spatial hierarchies and addresses CNN limitations.	Enhances accuracy and efficiency in plant disease classification and reduces computational overhead.	[110]
FPA	Optimizing plant phenotypic data and classification tasks.	Solves complex optimization problems through nature-inspired strategies.	Applied to optimize plant classification models and environmental data analysis.	[111]
AIS	Adaptive identification and optimization of plant data.	Mimics immune system for anomaly detection and pattern recognition.	Enhances species classification accuracy in complex environments through adaptive algorithms.	[112]
RF	Classification using complex plant traits and metabolite profiles.	Robust ensemble learning that mitigates overfitting.	Applied for classifying and clustering plant species from metabolic and environmental datasets.	[113]

NN, neural networks; SVM, support vector machines; DT, decision trees; RF, random forest; PCA, principal component analysis; KNN, k-nearest neighbors; LR, logistic regression; CNN, convolutional neural networks; FPA, flower pollination algorithm; AIS, artificial immune systems; RF, random forests.

**Table 4:** Case studies on nanoengineered devices for herbal medicine applications.

No.	Title	Year	Medicinal Plant	Technology/Technique	Outcome
1	Pandey and Ambwani [127]	2022	Ginseng	Nanoparticle-based sensors	Detected adulteration in ginseng products by identifying non-authentic species, supporting quality control
2	Hezekiah [128]	2021	Artemisia annua	Nano-fingerprinting techniques	Developed a nano-enabled framework to prevent misidentification of *Artemisia annua*
3	Munir et al. [129]	2020	Artemisia annua	Chemotaxonomic profiling and nanotechnology	Accurately distinguished *Artemisia annua* from similar species
4	Geetha, Sudha, and Praveena [126]	2023	Ginseng	Nano-biosensors	Enhanced accuracy in identifying *Panax* species, minimizing the risk of species substitution
5	Thiruvengadam, et al. [130]	2024	Various herbs	Nano-based biosensors and molecular analysis	Enabled detection of adulterated and counterfeit herbal products, ensuring consumer safety
6	Kumar [131]	2023	Various herbs	Nanotechnology for plant species identification	Demonstrated the efficacy of nanotechnology for rapid and accurate plant species identification
7	Singh and Yadav [132]	2024	Various herbs	Nano-based spectrometry and chemical analysis	Identified counterfeit herbs and quantified adulterants with high precision
8	Gasmi et al. [133]	2023	Ginseng	Nanoengineered delivery systems	Advanced understanding of the therapeutic properties of Ginseng through nanoengineered systems

## Data Availability

Not applicable.
